# Lymph microvascularization as a prognostic indicator in neuroblastoma

**DOI:** 10.18632/oncotarget.25457

**Published:** 2018-05-25

**Authors:** Irene Tadeo, Esther Gamero-Sandemetrio, Ana P. Berbegall, Marta Gironella, Félix Ritort, Adela Cañete, Gloria Bueno, Samuel Navarro, Rosa Noguera

**Affiliations:** ^1^ Pathology Department, Medical School, University of Valencia-INCLIVA, Valencia, Spain; ^2^ CIBERONC, Madrid, Spain; ^3^ Condensed Matter Physics Department, University of Barcelona, Barcelona, Spain; ^4^ CIBER-BBN, Madrid, Spain; ^5^ Hospital U I P La Fe, Valencia, Spain; ^6^ VISILAB, E.T.S.I. Industriales, University of Castilla-La Mancha, Ciudad Real, Spain

**Keywords:** lymphatic vessels, extracellular matrix, digital pathology, tumor interstitial fluid pressure, neuroblastoma

## Abstract

Neuroblastoma is the most common extra-cranial solid pediatric cancer and causes approximately 15% of all childhood deaths from cancer. Although lymphatic vasculature is a prerequisite for the maintenance of tissue fluid balance and immunity in the body, little is known about the relationship between lymphatic vascularization and prognosis in neuroblastoma. We used our previously-published custom-designed tool to close open-outline vessels and measure the density, size and shape of all lymphatic vessels and microvascular segments in 332 primary neuroblastoma contained in tissue microarrays. The results were correlated with clinical and biological features of known prognostic value and with risk of progression to establish histological lymphatic vascular patterns associated with unfavorable histology. A high proportion of irregular intermediate lymphatic capillaries and irregular small collector vessels were present in tumors from patients with metastatic stage, undifferentiating neuroblasts and/or classified in the high risk. In addition, a higher lymphatic microvascularization density was found to be predictive of overall survival. Our findings show the crucial role of lymphatic vascularization in metastatic development and maintenance of tumor tissue homeostasis. These patterns may therefore help to indicate more accurate pre-treatment risk stratification and could provide candidate targets for novel therapies.

## INTRODUCTION

The lymphatic system is essential for the maintenance of tissue fluid homeostasis, gastrointestinal lipid absorption, and immune trafficking. Whereas lymphatic regeneration occurs physiologically in wound healing and tissue repair, pathological lymphangiogenesis has been implicated in a number of chronic diseases such as lymphedema, atherosclerosis, and cancer [1]. Although lymphatics are well-known through histology, systematic lymphatic research only started with the identification of lymphatic endothelial markers [2–4]. Recently, Lund [5] showed that expression of lymphatic vessel markers such as *PDPN* and *LYVE1* may be predictive of immune microenvironments in melanoma. Additionally, the lymphatic system, previously thought to play a passive role in cancer metastasis, is now known to play an integral role in the metastatic spread of disease [6]. Moreover, tumor-associated inflammation and immunity critically depends on lymphatic vessel remodeling and drainage thus providing novel therapeutic targets in malignant disease [7]. Various studies of lymphatic vascularization in neuroblastoma (NB) have used different stainings, but focussed only on lymphatic density [8–10]. In these studies, lymphatic density was increased in NBs from patients with advanced-stage and in the high-risk group. Furthermore, until now, lymphatic vasculature in NB has been characterized through indirect quantitation [8, 9], and more accurate and standardized quantitation methods and stainings, such as those presented here, are needed.

Neuroblastoma is the most common extra-cranial solid pediatric cancer and is responsible for approximately 15% of all childhood cancer deaths [11, 12]. Prognosis of NB is dependent on clinical parameters such as age, stage, histopathologic category and genetic status [13]. Adverse prognostic factors include age ≥18 months at diagnosis, undifferentiated and poorly differentiated histopathology and specific genetic abnormalities, such as *MYCN* oncogene amplification (MNA) and segmental chromosomal aberrations (SCA) [11]. The majority of patients with adverse NBs have widespread lymphatic and/or hematogenous metastases at diagnosis, and while angiogenesis has been studied extensively [14–16], few reports have examined lymphangiogenesis in NB [8, 17]. Despite the improvement in survival through the application of a well-defined pre-treatment risk classification [18], this classification remains unsuccessful with standard first-line treatment in most high-risk cases [19]. Therefore, urgent advances in research on new pre-treatment stratification factors and new therapeutic targets are needed to improve survival and long-term quality of life [20, 21].

Recently, in addition to vascular density, other parameters such as the size and shape of the microvessels, have been shown to be significantly related to prognosis for blood vascularization in NB [15] and in other malignancies [22, 23]. In this regard, the present study presents the application of an automated tool for objective quantitation of tissue vascularization [15, 16] to assess whether the abundance and morphologic features of lymphatic vessels are related to prognosis in NB and could therefore be used as a factor to enhance the pre-treatment risk stratification or may provide therapeutic targets.

## RESULTS

### Quantification of the lymphatic vessels

**Figure 1:**
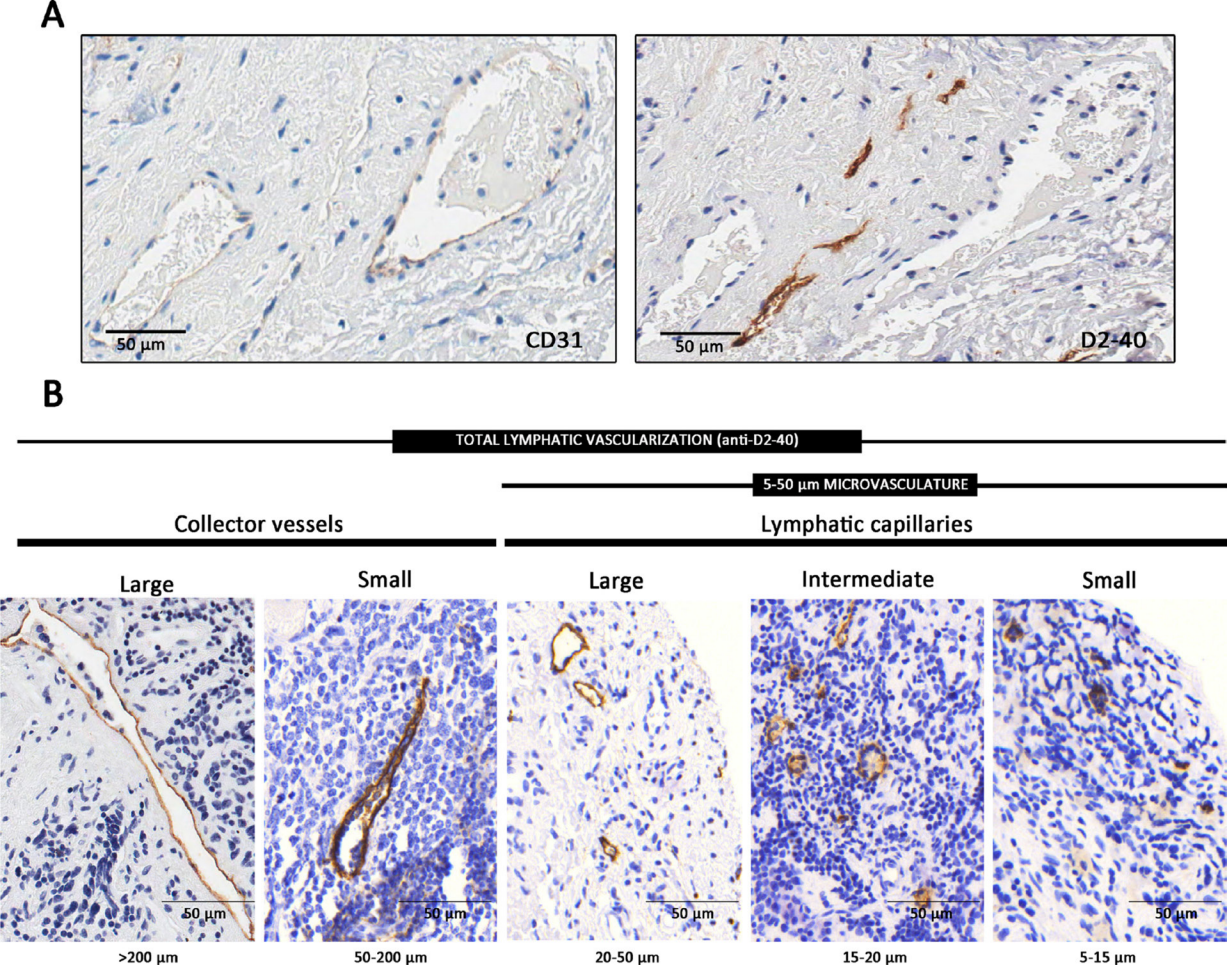
Lymphatic vessels (**A**) Complementary staining of lymphatic vessels, with immunohistochemistry anti-D2-40 and of blood vessels with anti-CD31. (**B**) Types of lymphatic vessels stained with anti-D2-40 immunohistochemistry in neuroblastoma samples. Lymphatic capillaries are thin-walled tubes formed of a single layer of superposed endothelial cells with button-like junctions between them. The lymphatic capillaries are joined together to form collector vessels (lymphatic vessels of greater thickness). The intrinsic design of the TMAs avoided areas with huge vascular structures so very few samples presented large collector vessels in their ECM (0.1% of all present blood vessels, respectively), thus these structures were not considered.

332 samples included in the tissue microarrays (TMAs) were evaluable (cylinders preserved during processing, no artefacts, representative tissue, and sufficient material). Anti-D2-40 immunoreactivity was observed in 206 of these samples (62.1%) (Figure 1A). Figure 1B shows the types of lymphatic vessels that stained with anti-D2-40. In general, NB tumors had a median density of 40.6 vessels/mm^2^ (range 1–979.4), 75% corresponding to small capillaries. The distribution according to size of all detected lymphatic vessels together with the statistical descriptors of density and the variables that provided statistically significant results are presented in Table 1. The statistical descriptors of the remaining variables are shown in Supplementary Table 1.

**Table 1:** Distribution of lymphatic vascularization descriptors with statically significant results

Lymphatic vessel class	Parameter		Median	Mean	Range	SD
**5–15 μm** *n*: 206 (99.5%)	Quantity	Density	31.13	80.84	0.54–712.9	124.77
	Size	Area	32.60	32.20	16.93–58.71	6.48
		Width	5.28	5.28	3.35–7.42	0.69
		Perimeter	25.87	25.60	17.98–40.29	2.79
	Shape	Branching	2.38	2.38	2.00–40.29	0.21
**15–20 μm** *n*: 185 (89.4%)	Quantity	Density	4.30	11.73	0.42–127.41	19.20
		SA	0.04	0.10	0.00–1.23	0.18
		Rel. density	9.09	10.20	1.45–50.0	5.81
		Rel. SA	9.86	12.43	0.23–89.80	11.79
	Shape	Roundness	2.73	2.75	1.53–5.67	0.50
**20–50 μm** *n*: 183 (88.4%)	Quantity	Density	6.39	16.64	0.34–154.04	25.78
**50–200 μm** *n*: 138 (66.6%)	Quantity	Density	2.35	4.81	0.24–25.68	5.51
	Size	Length	74.99	76.90	50.61–134.73	17.38
		Perimeter	301.60	301.60	132.05–655.74	106.21
	Shape	Roundness	8.35	8.35	2.90–21.18	3.02
		Shape factor	1.92	9.00	0.24–255.86	1.92
		Branching	2.35	4.81	0.24–25.68	5.51
**TOTAL** *n*: 207 (100%)	Quantity	Density	40.63	109.01	1.0–979.43	170.05

Distribution of all detected lymphatic vessels according to their morphometric variables and statistical descriptors associated with statistically significant results are shown. Only a few samples presented lymphatic macrovascularization (>200 μm), so this class was not considered.

5–15 μm: small capillaries; 15–20 μm: intermediate capillaries; 20–50 μm: large capillaries; 50–200: small collector vessels. *n*: number of samples presenting lymphatic vessels of a given subgroup, SD: Standard deviation. SA: stained area, Rel: relative. Units: Density (n/mm^2^), Area (μm^2^), Width (μm), Perimeter (μm), Branching (units), Rel. density (%), SA (%), Length (μm), Roundness (no units).

### Abundant small and intermediate capillaries and irregular and large lymphatic vessels corresponding to small collectors are associated with poor prognostic factors

The following features were identified: NB with undifferentiated or poorly differentiated phenotype presented wider small capillaries with more branching than NB with differentiating neuroblasts occupying a large area in the tumor. A high proportion of intermediate capillaries were present in tumors from patients with metastatic stage, >18 months, MNA and/or classified as high-risk, the capillaries were more irregular in NB without differentiating cells. Irregular small collector vessels were associated with metastatic stage, >18 months, undifferentiated NB and high risk. The *p*-values for all morphometric variables of all lymphatic vessel microvascularization classes related with the INRG pretreatment risk stratification factors are shown in Table 2.

**Table 2:** *p*-values and nature of the relationship between lymphatic vascularization and the INRG poor-prognostic factors

Lymphatic vessel class	Parameter		Stage: M	Age: >18 months	Histopath.: (pd, uNB)^*^	*MYCN:* MNA	11q: 11qD	Risk group: high-risk
**5–15 μm**	Quantity		−	−	−	−	−	−
	Size	Area	−	−	0.022 ↑	−	−	−
		Width	−	−	0.012 ↑	−	−	−
		Perimeter	−	−	0.046 ↑	−	−	−
	Shape	Branching	−	−	0.024 ↑	−	−	−
**15–20 μm**	Quantity	Density	−	0.006 ↑	−	−	−	0.020 ↑
		%SA	−	0.045 ↑	−	−	−	−
		Rel. density	0.011 ↑	−	−	0.006 ↑	−	0.003 ↑
		Rel. %SA	0.001 ↑	−	−	−	−	−
	Size		−	−	−	−	−	−
	Shape	Roundness	−	−	0.013 ↑	−	−	−
**20–50 μm**			−	−	−	−	−	−
**50–200 μm**	Quantity		−	−	−	−	−	−
	Size	Length	−	0.022 ↑	−	−	−	−
		Perimeter	−	0.009 ↑	−	−	−	−
	Shape	Roundness	0.025 ↑	0.002 ↑	0.046 ↑	−	−	0.006 ↑
		Shape factor	−	−	0.008 ↑	−	−	−
		Branching	0.044 ↓	−	−	−	−	−
TOTAL			−	−	−	−	−	−

Only morphometric variables, for all lymphatic microvascularization classes, with a statistically significant relationship with pre-treatment risk stratification factors are shown.

5–15 μm: small capillaries; 15–20 μm: intermediate capillaries; 20–50 μm: large capillaries; 50–200: small collector vessels. %SA: percentage of stained area, Rel: relative, M: metastatic, Histopath: histopathology, pdNB: poorly differentiated neuroblastoma, uNB: undifferentiated neuroblastoma, 11qD: 11q deletion, -: not statistically significant, ↑/↓: higher or lower median value for the poor-prognostic group(s). ^*^There are no statistically significant differences between pdNB and uNB.

### Lymphatic vessel morphometric variables can be combined to predict the risk pre-treatment stratification group

A binary logistic regression analysis was performed to combine the different lymphatic vessel morphometric variables to predict risk (high risk or non-high risk). The lowest values of the median were taken as a reference category and the coefficients Exp(B) indicated an increase or decrease in high-risk susceptibility (Table 3).

**Table 3:** Logistic regression of lymphatic vessel morphometric variables

Significant lymphatic vessel parameter	B	S.E	Wald	*p*-value	Exp(B)
**5–15 μm Rel. %SA** **(high values)**	–2.173	0.810	7.197	0.007	0.114
**15–20 μm Width** **(high values)**	–1.706	0.810	4.435	0.035	0.182
**15–20 μm Rel. %SA** **(high values)**	–1.778	0.687	6.700	0.010	5.916
^*^15–20 μm Rel. density **(high values)**	0.966	0.537	3.23	0.072	2.628
**20–50 μm Width** **(high values)**	–1.644	0.652	6.349	0.012	0.193
**20–50 μm Roundness** **(high values)**	–1.404	0.508	7.644	0.006	4.072
**20–50 μm Rel. density** **(high values)**	–1.310	0.647	4.096	0.043	0.270
**TOTAL Branching** **(high values)**	–1.509	0.646	5.454	0.020	0.221

Significant lymphatic vessel parameters that predict high-risk with *p*-value < 0.05. ^*^p-value < 0.1. SA: stained area, Rel: relative. S.E: Standard Error. Coefficients Exp (B)<1 indicate that high values of this parameter reduce high-risk susceptibility. Coefficients Exp (B)>1 indicate that high values of this parameter increase high-risk susceptibility.

5–15 μm: small capillaries; 15–20 μm: intermediate capillaries; 20–50 μm: large capillaries.

The logistic equation can be written as:

0.114^5–15 μmRel.SA^ × 0.182^15–20 μmWidth^ × 5.916^15–20 μmRel.SA^ × 0.193^20–50 μmWidth^ × 4.072^20–50 μmRoundness^ × 0.270^20–50 μmRel.density^ × 0.221^TotalBranching^

**Figure 2:**
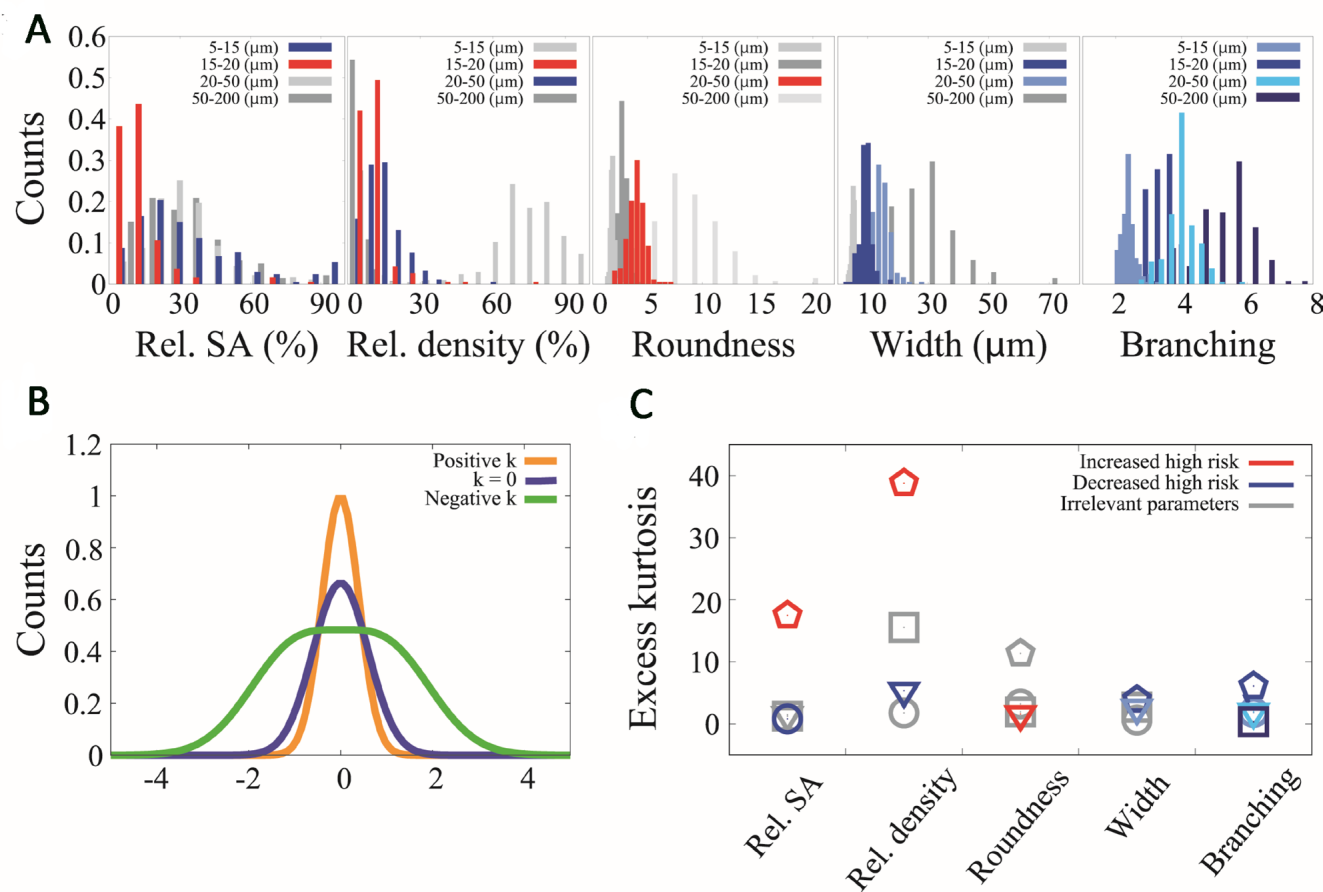
Histograms and kurtosis analysis for parameters related with high risk (**A**) Histograms of relative stained area (Rel. SA), relative density (rel. density), roundness, width and branching for all the different vessel calibers (5–15 μm, 15–20 μm, 20–50 μm, 50–200 μm). In red we represent the parameter distributions that in high values increase the probability of being high risk, in blue the parameter distributions that in high values decrease the probability of being high risk and in grey the parameter distributions that their values are not related to high risk. (**B**) Schematic graph that relates the shape of three different distributions with the value of their kurtosis. (**C**) Kurtosis comparison of the distributions of (A) where each column represents one histogram. The color criteria remain the same as (A) and the different vessel calibers are labeled with different point types where circles represent 5–15 (μm), pentagons represent 15–20 (μm), triangles represent 20–50 (μm) and squares represent 50–200 (μm).

According to this equation, lymphatic vessel impact on risk was: i) High values of relative %SA, relative density of intermediate capillaries and roundness of large capillaries increased six, three and four times, respectively, the probability of being high risk. Thus, the presence of more abundant intermediate capillaries and irregular large capillaries indicated predisposition to high-risk NB disease. ii) High values of small microvascularization (5–15 μm) relative %SA, 15–20 μm width, 20–50 μm width, 20–50 μm relative density and total branching reduced the probability of being high risk by around 80% for each parameter. Therefore, a wider lymphatic microvascularization (15–50 μm) with more branching is a better predictor of being high risk. Histograms for parameters related to the high risk are shown in Figure 2A. A relevant feature of the data is the large heterogeneity characterizing the different parameters across the primary tumors where the SD is comparable to the mean. Such broad heterogeneity produces statistical distributions that strongly deviate from a normal distribution. In Figure 2B and 2C we show the kurtosis (which quantifies deviations from the normal distribution) for some parameters across the lymph level classes related to the high risk. Our data show that except for small branching collector vessels with a value of Zg2≈1, all evaluated variables presented a value of Zg2 in the range [2.4–114.1]. Interestingly the variables that belong to the high-risk group seem to present the largest excess kurtosis values, suggesting that excess kurtosis in these variables might be a potential indicator of risk.

### Abundant lymphatic microvascularization is related with poor survival

**Figure 3:**
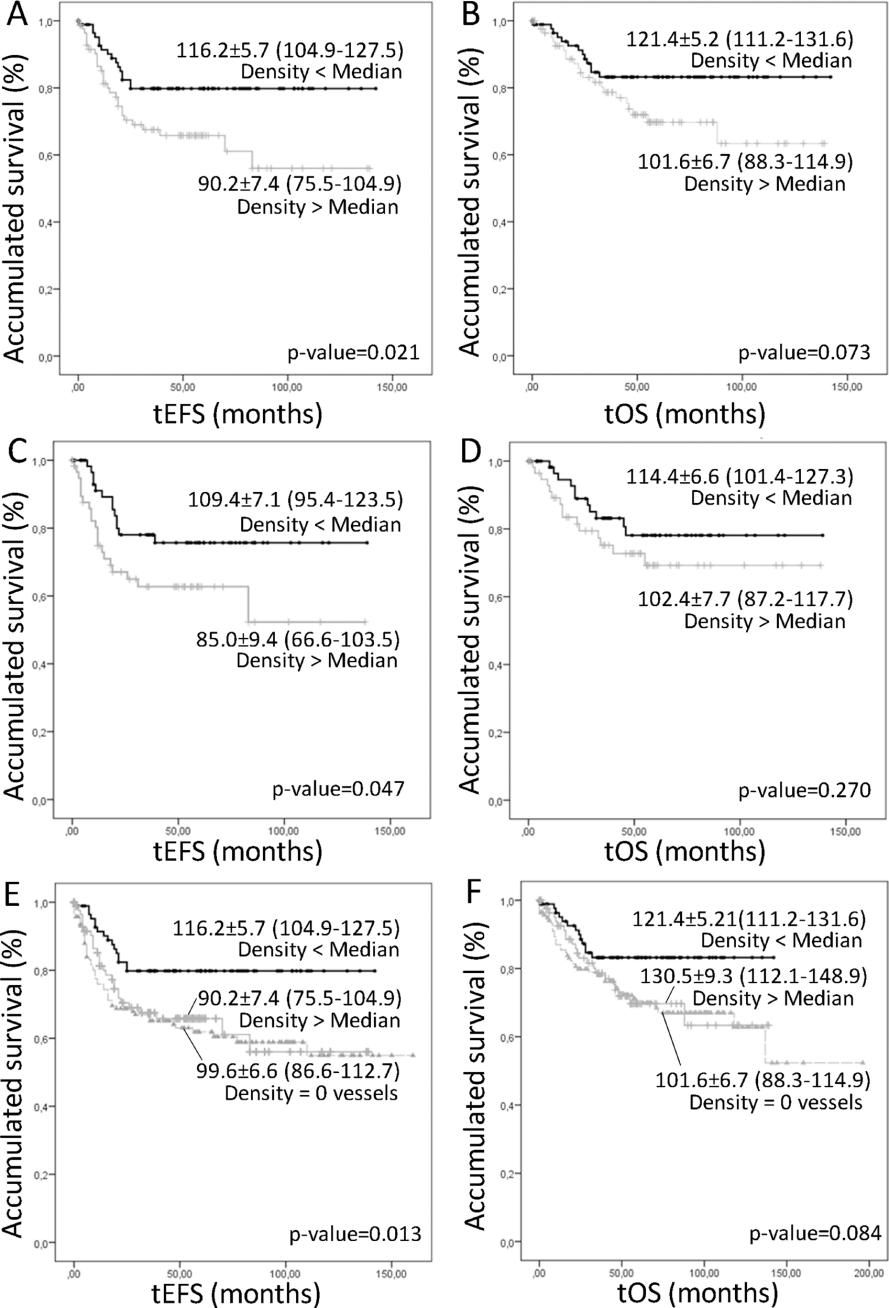
Kaplan–Meier graphs showing the different accumulated EFS (**A**, **C**, **D**) or OS (**B**, **D**, **F**) depending on different variables. *P*-values and survival rates are shown. (A–B) Intermediate lymphatic capillaries over or under the median. (C–D) Lymphatic microvascularization (small, intermediate and large capillaries, together) over or under the median. (**E**–F) Comparison of the survival of the patients with samples not presenting lymphatic vascularization and presenting a number of intermediate capillaries over and under the median.

**Figure 4:**
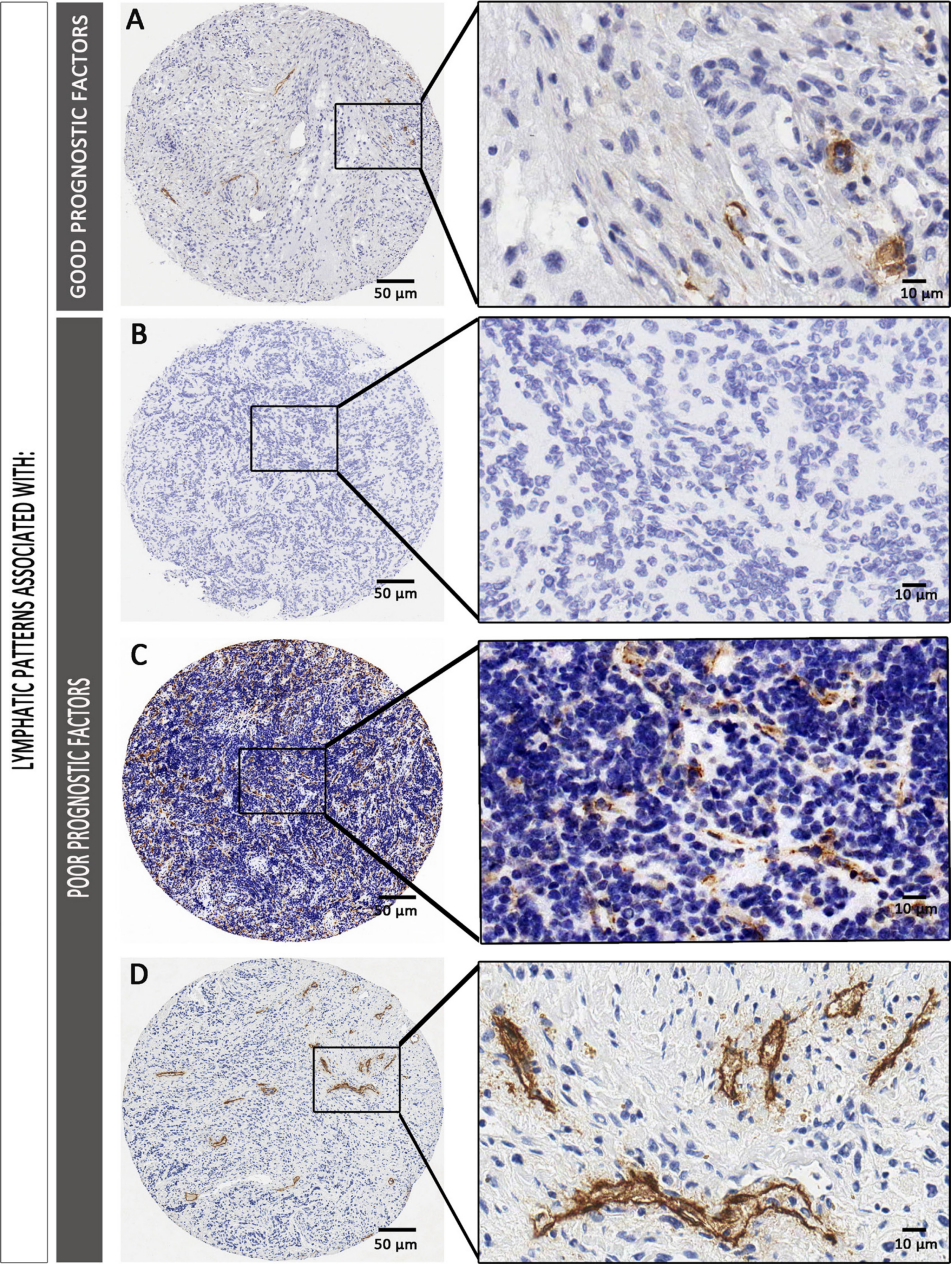
Examples of lymphatic patterns in neuroblastoma with favorable (**A**) and unfavorable prognostic factors (**B**–**D**). (A) Sample corresponding to a favorable NB (dNB). Differentiated histology can be appreciated, with few lymphatic vessels, mostly corresponding to round large capillaries and collector vessels. (B–D) Samples corresponding to an unfavorable sample B. without lymphatic vessels. (C) with abundant irregular intermediate capillaries and D. with irregular small collector vessels.

A higher density of lymphatic vessels corresponding to the intermediate capillaries was related to poorer event-free survival (EFS) (5-year EFS% of 65.8 ± 5.5), and tended to be related to lower overall survival (OS), compared to that of patients with samples presenting a lower lymphatic vessel density (5-year EFS% 79.8 ± 4.5) (Figure 3A, 3B). We also tested the relationship between microvascularization and survival by combining all lymphatic vessels found in any of the microvascularization classes (from 5 to 50 μm) and dichotomizing this new variable using the median value as the cut off. We still found that an increased lymphatic microvascularization was related with poorer prognosis (5-year EFS% of 62.7 ± 6.7 *versus* 75.7 ± 5.9 for lower lymphatic microvascularization) (Figure 3C, 3D). Additionally, the influence of the absence of lymphatic vascularization on survival was also tested, showing that patients presenting these samples had the same poor prognosis as patients whose samples presented many intermediate capillaries (5-year EFS% of 61.9 ± 4.5) (Figure 3E, 3F). Examples of the lymphatic patterns in neuroblastoma with favorable and unfavorable factors are shown in Figure 4.

When considering all INRG variables together with total lymphatic vascularization, a Cox regression analysis demonstrated that a higher microvascularization density was still found to influence EFS (Exp(B): 4.547; Hazard ratio 95% Confidence interval (CI): 2.403 (1.073–5.381); *p*-value: 0.033), after metastatic stage (Exp(B): 11.280; Hazard ratio (95% CI): 4.703 (1.906–11.608); *p*-value: 0.001), MNA (Exp(B): 5.057; Hazard ratio (95% CI): 2.662 (1.134–6.251); *p*-value: 0.025), and age >18 months (Exp(B): 4.809; Hazard ratio (95% CI): 3.032 (1.125–8.171); *p*-value: 0.028). 11q status and histopathology were excluded from the equation (*p*-value > 0.05). OS was only influenced by the INRG variables of stage, *MYCN* status and age.

## DISCUSSION

Several studies have revealed the importance of lymphatic vascular density in the prognosis of different malignancies [24–27]. The application of digital pathology methods to TMA samples allows data to be collected and translated into biological significance. Our results show that tumors from patients with unfavorable independent prognostic INRG variables contain mostly intermediate capillaries and irregular small collector vessels. In addition, an increment in total lymphatic microvascularization was related with poorer prognosis, information which may be helpful to further stratify patients.

Tissue vascularization has usually been described following three different quantitative methods (subjective, objective non-morphometric and morphometric methods), based on the detection of the differential staining of the vessels with more or less specific techniques (immunohistochemistry against factor VIII related von Willebrand factor [28], CD34 [29], CD31 [15], caveolin [30] or CD105 [31] for blood vessels; against D2-40 [32], PROX1 [10] and LYVE-1 [5] for lymphatic vessels and traditional histology methods such as the combination of H&E and Victoria blue-Van Gieson stainings, to reliably distinguish lymphatic channels from small blood vessels [33]) on whole slides and/or in TMAs slides. When evaluating microvascularization by subjective vascular grading, the number of microvessels is assessed manually with a microscope in the most active regions of vascularization (hot-spots), in small fields. Microvessel density is also graded subjectively from 1 to 4+ [34, 35]. Objective non-morphometric Chalkley point counting is an indirect estimate of the relative microvascular area that uses a 25-point eyepiece graticule applied to three subjectively-chosen hot-spots from each tumor section. [35, 36]. Other size and shape-related parameters such as length, perimeter, roundness and branching are commonly associated with morphometric methods. In general, a moderate negative correlation exists between microvascular density and vessel size-related parameters such as total microvascular area, length and perimeter [37]. We used the previously published custom-designed tool [15, 16] to close open-outline vessels in order to measure morphometric parameters and properly quantify the vascular density in the different microvascular supply segments. Although TMAs do not normally include areas of invasion or large vessels, for our study, we selected areas that better represent the NB histology. Furthermore, at least two cylinders representing each sample were quantified, thus minimizing the effect of tumor heterogeneity. This procedure potentially enables the results of our work to be extrapolated to other cohorts. Since peritumoral vascular invasion may be predictive of distant metastasis [38, 39], future studies on complete tumor sections would be of interest.

The aberrant growth of regional lymphatic vessels is associated with enhanced locoregional metastasis and poor outcome in many solid tumors [40]. Lymphatic drainage from the tumor probably plays multiple and complex roles in tumor progression. Lymphatic vessels carry fluid and immune cells from peripheral tissues to draining lymphatic nodes, where both components help shape immunity and maintain tolerance to autoantigens [41–43]. Without lymphatic vessels and their associated drainage, peripherally activated dendritic cells cannot activate immune response, which could explain the poor prognosis associated with the absence of lymphatic vessels in our cohort [44]. Similarly, our results correlate high lymphatic microvascularization with poor prognosis. Specifically, a high proportion of intermediate capillaries and irregular large capillaries indicated a predisposition towards high risk. In addition, higher density of lymphatic microvessels and, specifically, of intermediate capillaries, was related to poorer EFS. Furthermore, the influence of a high microvascularization density was preserved when considering the INRG prognostic factors, having an impact similar to that of age >18 months. Recently, we described that a high plexiform blood microvascularization is required to maintain tissue homeostasis [15]. Specifically, larger and rounder capillaries (5–15 μm) and sinusoid-enriched vessels (20–50 μm) can contribute to an increase in intratumoral pressure and chaos within the tumor tissue. Moreover, this blood vessel morphology was present in tumor tissue of NB patients with INRG poor prognostic factors. Based on the results for lymphatic vascularization, we hypothesize that a specific microvascularization (blood and lymphatic vessels) is needed to maintain tumor tissue homeostasis. Indeed, the same poor-prognosis samples correlated with high lymphatic microvascular density and irregular small collector vessels, on the one hand, as well as with large blood capillaries and high density of sinusoids in tumor tissue, on the other. In fact, this enriched tumor vessel morphology may cause an increased interstitial fluid pressure which has already been identified as a factor that can impede effective cancer treatment [5, 45]. We also observed that the absence of lymphatic vessels was related to low overall survival. We hypothesize that, as with blood vessels [46], cancer cells could mimic endothelial cells forming lymphatic channels and contribute to poor prognosis in NB.

Multiple antiangiogenic therapeutic strategies have been developed in the last decades for many different malignancies [47]. Several direct angiogenic inhibitors of endothelial cell functions and indirect anti-angiogenic agents that block the production or activity of pro-angiogenic molecules have been shown to reduce tumor interstitial fluid pressure [48, 49]. However, inhibition of angiogenesis causes hypoxia in tumors which provokes the overexpression of lymphangiogenic factors, enhancing tumor metastasis through lymphatics [50, 51]. Thus, lymphangiogenesis has recently emerged as a suitable therapeutic target to block metastases. Drugs such as Tivantinib, Onartuzumab, Rilotumumab, Trebananib, IMC-3C5, and AD0157 have been used to block this crucial event by targeting the VEGF-C/VEGFR-3 signaling axis, the most relevant and specific pathway that promotes lymphangiogenesis in pathological situations [52]. Moreover, several studies have focused on the value of normalizing tumor vasculature to improve response to conventional anticancer therapies [5, 53]. This normalized tumor vasculature becomes less permeable and tortuous and leads to reduced fluid extravasation within the interstitium, resulting in a decrease in tumor interstitial fluid pressure [54, 55].

In conclusion, NB is characterized by a specific lymphatic microvascularization pattern. In addition to vessel density, the morphometric parameters of shape and size are also associated with unfavorable prognostic variables and could be taken into account to enhance pre-treatment risk stratification. These findings, together with other extracellular matrix parameters, are helping to define new strategies based on vascular normalization and targeted therapy. Given the existence of a characteristic blood microvascular pattern [15] and our results, which demonstrate a specific lymphatic microvascularization pattern in tumor tissue, we suggest considering an innovative strategy in cancer treatment by combining anti-lymphangiogenic and anti-angiogenic drugs to obtain normalized tumor vasculature.

## MATERIALS AND METHODS

### Samples

Material from 332 primary NB tumors (at least two representative cylinders of 1mm of each tumor) were included TMAs, comprising poorly differentiated NB (pdNB, *n* = 274), undifferentiated NB (uNB, *n* = 60), NOS (*n* = 41) and differentiating NB (dNB, *n* = 37), histopathologically categorized following the International Neuroblastoma Pathology Classification (INPC) [56]. All samples were from patients referred to the Spanish Reference Centre for NB Biological and Pathological studies (Department of Pathology, University of Valencia-INCLIVA) from 1996 to 2007. These patients were classified into risk groups (high risk versus non-high risk) according to the stage of the disease (metastatic versus non-metastatic), the age of the patients (>18 months versus ≤18 months), the histopathologic differentiation (pdNB and uNB versus dNB; NOS tumor patients were excluded from the statistically analyses) and the genetic status of *MYCN* oncogene (amplified, MNA versus non-amplified, MNNA) or the 11q chromosome arm (deleted, 11qD versus non-deleted, 11qND), following the International NB Risk Group (INRG) classification [18]. The status of these prognostic factors was determined in accordance with previously-published guidelines [57–61] and the studied cohort was shown to behave as expected regarding the relationship of all INRG prognostic factors and outcome, with *p*-values = 0.000 in all cases.

Histologic and genetic studies were approved by the Spanish Society of Pediatric Hematology and Oncology (file number: 59C18ABR2002), and the European Committee (file number: 2010-021396-81), as well as by the Ethical Committee of the University of Valencia (file numbers: H1270128195640 and A1420714159483). Participants or their family members/legal guardians provided written informed consent for histological and genetic studies performed in our laboratory. Clinical data were provided by the pediatric oncologists in charge or by the Reference center for NB clinical studies, including outcome information (EFS, defined as the length of time from the date of diagnosis to any progression of the disease; and OS, defined as the length of time from the date of diagnosis for patients who are still alive).

### Immunohistochemistry

One 3μm section of each TMA was cut and immunostained with anti-D2-40 monoclonal antibody (1/40) (Dako, mouse anti-human) that stains podoplanin, a transmembrane mucoprotein selectively expressed in lymphatic vessel endothelium (Figure 1A).

### Image analysis

All immunostained slices were digitized with the whole-slide scanner Aperio SlideScan XT (Aperio technologies). Individual images of all TMA cylinders were exported by the Aperio ImageScope in tiff format. Histologically, it has been described that the size of lymphatic capillaries in normal tissue ranges from 10–150 μm and the lymphatic collectors measure between 150 μmand 2 mm [62]. In tumors however, lymphatic vessels have been shown to be narrow and to present a thickening of their walls [63, 64]. The Angiopath^®^ tool used here measures the internal lumen of the vessels regardless of their wall thickness. Moreover, we had previously analyzed the HE and Masson's trichrome stainings of the studied cohort [65] and observed that the size of the lymphatic vessels in the tumor tissue could be correlated with that of the blood vessels. The previous findings (narrow lymphatic vessels, internal lumen measurement and observed size compared to that of blood vessels) led us to define smaller sizes for neuroblastic tumor lymphatics, measured with Angiopath^®^: 5–50 μm for capillaries (small capillaries 5–15 μm, intermediate capillaries 15–20 μm, large capillaries 20–50 μm) and 50–200 μm for collectors (small collector vessels 50–200 μm and large collector vessels >200 μm) (Figure 1B). For each lymphatic segment, the following parameters were measured: 1. Quantity (percentage of stained area (%SA); density (n/mm^2^) and relative %SA (rel. %SA) and relative density (rel. density); 2. Size (area, length, width, perimeter) and 3. Shape (roundness, aspect, perimeter ratio, deformity, shape factor and branching), as previously reported by our group for blood vascularization [15]. The classification into groups was made according to the longest axis of each vessel, regardless of the orientation, which given the size of the cohort is assumed to be random and with a negligible effect on the results.

### Statistical methods

In all cases, a significance level of 95% was established and the SPSS statistical analysis software (version 22) was used. Non-evaluable samples (cylinders lost during processing, artefacts, non-representative tissue, and scant material) were excluded from the analyses. Samples with no immunoreactivity against D2-40 were also excluded from the statistical analysis except for a subsection of the survival analysis.

The numerical continuous variables of density, size and shape from all lymphatic vessel classes derived from the morphometric analysis did not follow a normal distribution, therefore the non-parametric Mann–Whitney test was used to related these variables with the prognostic INRG categories. The continuous morphometric variables were dichotomized using the median, as it is the most accurate statistical descriptor for that purpose. A binary logistic regression was performed, using the back elimination method (conditional), combining all lymphatic vessels morphometric variables and relating them with the risk group.

Survival analyses were performed using Kaplan–Meier curves and log-rank test. The dichotomized variables related to the density of the different lymphatic vessel classes and the total density of lymphatic microvascularization (small, intermediate and large capillaries, together) were used. Additionally, the strength of the relationship of total lymphatic microvascularization with survival was tested using Cox survival regression using Wald (step back) test, including all INRG prognosis-related variables.

For histogram analysis, we analyzed an excel file *VL.xlxs* containing all the parameters extracted from the histologies of 332 different patients of primary tumor respect to the lymphatic vessels. Each column contained the data associated with one parameter in a particular range of vessels calibers and the total microvasculature (5–50 μm). Using Rice's Rule for choosing the binning (number of bins = 2N¹/^3^, where N is the size of the column vector), we computed a histogram for each column and we have normalized it dividing the counts of each bin for the total number of counts. In the specific cases of the parameters relative stained area and relative density, we have discarded the 0 values which indicate no vessels in the histology image.

Kurtosis analysis is an estimator of the normality of the distribution and it is defined as the fourth standardized moment,

$$\text{Kurt}(X)=E[{\left(\frac{X-\mu }{\sigma }\right)}^{4}]$$

where *E* is the expected value, *X* is the experimental data, μ is the mean value and σ is the standard deviation. In our analysis we used another possible definition: Excess Kurtosis, where *Excess Kurtosis = 0* corresponds to the normal distribution,

ExcessKurtosis(X)=Kurt(X)−3

The statistical significance of excess kurtosis G2 is defined by Zg2=G2/SEK where SEK is the standard error of kurtosis (≈ 0.34). This estimator defines a leptokurtic distribution with a significance of 95% whenever Zg2 > 2. The range −2< Zg2 < 2 defines a normal distribution with a significance of 95%.

## SUPPLEMENTARY MATERIALS FIGURES AND TABLES





## References

[1] Jiang X, Nicolls MR, Tian W, Rockson SG (2018). Lymphatic Dysfunction, Leukotrienes, and Lymphedema. Annu Rev Physiol.

[2] Kahn HJ, Bailey D, Marks A (2002). Monoclonal antibody D2-40, a new marker of lymphatic endothelium, reacts with Kaposi's sarcoma and a subset of angiosarcomas. Mod Pathol.

[3] Banerji S, Ni J, Wang SX, Clasper S, Su J, Tammi R, Jones M, Jackson DG (1999). LYVE-1, a new homologue of the CD44 glycoprotein, is a lymph-specific receptor for hyaluronan. J Cell Biol.

[4] Breiteneder-Geleff S, Soleiman A, Kowalski H, Horvat R, Amann G, Kriehuber E, Diem K, Weninger W, Tschachler E, Alitalo K, Kerjaschki D (1999). Angiosarcomas express mixed endothelial phenotypes of blood and lymphatic capillaries: podoplanin as a specific marker for lymphatic endothelium. Am J Pathol.

[5] Lund AW, Wagner M, Fankhauser M, Steinskog ES, Broggi MA, Spranger S, Gajewski TF, Alitalo K, Eikesdal HP, Wiig H, Swartz MA (2016). Lymphatic vessels regulate immune microenvironments in human and murine melanoma. J Clin Invest.

[6] Makinen T, Norrmen C, Petrova TV (2007). Molecular mechanisms of lymphatic vascular development. Cell Mol Life Sci.

[7] McAllaster JD, Cohen MS (2011). Role of the lymphatics in cancer metastasis and chemotherapy applications. Adv Drug Deliv Rev.

[8] Ramani P, Dungwa JV, May MT (2012). LYVE-1 upregulation and lymphatic invasion correlate with adverse prognostic factors and lymph node metastasis in neuroblastoma. Virchows Arch.

[9] Ramani P, Somerville MS, May MT (2012). Podoplanin lymphatic density and invasion correlate with adverse clinicopathologic and biological factors and survival in neuroblastomas. Am J Surg Pathol.

[10] Ramani P, Norton A, Somerville MS, May MT (2012). PROX1 lymphatic density correlates with adverse clinicopathological factors, lymph node metastases and survival in neuroblastomas. J Neurooncol.

[11] Maris JM, Hogarty MD, Bagatell R, Cohn SL (2007). Neuroblastoma. Lancet.

[12] Kaatsch P (2010). Epidemiology of childhood cancer. Cancer Treat Rev.

[13] Maris JM, Matthay KK (1999). Molecular biology of neuroblastoma. J Clin Oncol.

[14] Rossler J, Taylor M, Geoerger B, Farace F, Lagodny J, Peschka-Suss R, Niemeyer CM, Vassal G (2008). Angiogenesis as a target in neuroblastoma. Eur J Cancer.

[15] Tadeo I, Bueno G, Berbegall AP, Fernandez-Carrobles MM, Castel V, Garcia-Rojo M, Navarro S, Noguera R (2016). Vascular patterns provide therapeutic targets in aggressive neuroblastic tumors. Oncotarget.

[16] Fernandez-Carrobles MM, Tadeo I, Bueno G, Noguera R, Deniz O, Salido J, Garcia-Rojo M (2013). TMA vessel segmentation based on color and morphological features: application to angiogenesis research. ScientificWorldJournal.

[17] Kashima K, Watanabe M, Satoh Y, Hata J, Ishii N, Aoki Y (2012). Inhibition of lymphatic metastasis in neuroblastoma by a novel neutralizing antibody to vascular endothelial growth factor-D. Cancer Sci.

[18] Cohn SL, Pearson AD, London WB, Monclair T, Ambros PF, Brodeur GM, Faldum A, Hero B, Iehara T, Machin D, Mosseri V, Simon T, Garaventa A (2009). The International Neuroblastoma Risk Group (INRG) classification system: an INRG Task Force report. J Clin Oncol.

[19] Pui CH, Gajjar AJ, Kane JR, Qaddoumi IA, Pappo AS (2011). Challenging issues in pediatric oncology. Nat Rev Clin Oncol.

[20] Pinto NR, Applebaum MA, Volchenboum SL, Matthay KK, London WB, Ambros PF, Nakagawara A, Berthold F, Schleiermacher G, Park JR, Valteau-Couanet D, Pearson AD, Cohn SL (2015). Advances in Risk Classification and Treatment Strategies for Neuroblastoma. J Clin Oncol.

[21] Henssen AG, Odersky A, Szymansky A, Seiler M, Althoff K, Beckers A, Speleman F, Schafers S, De Preter K, Astrahanseff K, Struck J, Schramm A, Eggert A (2017). Targeting tachykinin receptors in neuroblastoma. Oncotarget.

[22] Korkolopoulou P, Patsouris E, Kavantzas N, Konstantinidou AE, Christodoulou P, Thomas-Tsagli E, Pananikolaou A, Eftychiadis C, Pavlopoulos PM, Angelidakis D, Rologis D, Davaris P (2002). Prognostic implications of microvessel morphometry in diffuse astrocytic neoplasms. Neuropathol Appl Neurobiol.

[23] Laitakari J, Nayha V, Stenback F (2004). Size, shape, structure, and direction of angiogenesis in laryngeal tumour development. J Clin Pathol.

[24] Ullah E, Nagi AH, Lail RA (2012). Angiogenesis and mast cell density in invasive pulmonary adenocarcinoma. J Cancer Res Ther.

[25] Haldorsen IS, Stefansson I, Gruner R, Husby JA, Magnussen IJ, Werner HM, Salvesen OO, Bjorge L, Trovik J, Taxt T, Akslen LA, Salvesen HB (2014). Increased microvascular proliferation is negatively correlated to tumour blood flow and is associated with unfavourable outcome in endometrial carcinomas. Br J Cancer.

[26] Ozerdem U, Wojcik EM, Duan X, Ersahin C, Barkan GA (2013). Prognostic utility of quantitative image analysis of microvascular density in prostate cancer. Pathol Int.

[27] Barau A, Ruiz-Sauri A, Valencia G, Gomez-Mateo Mdel C, Sabater L, Ferrandez A, Llombart-Bosch A (2013). High microvessel density in pancreatic ductal adenocarcinoma is associated with high grade. Virchows Arch.

[28] Sabarinath B, Sriram G, Saraswathi TR, Sivapathasundharam B (2011). Immunohistochemical evaluation of mast cells and vascular endothelial proliferation in oral submucous fibrosis. Indian J Dent Res.

[29] Zygon J, Szajewski M, Kruszewski WJ, Rzepko R (2017). VEGF, Flt-1, and microvessel density in primary tumors as predictive factors of colorectal cancer prognosis. Mol Clin Oncol.

[30] Mohammed DA, Helal DS (2017). Prognostic significance of epithelial/stromal caveolin-1 expression in prostatic hyperplasia, high grade prostatic intraepithelial hyperplasia and prostatic carcinoma and its correlation with microvessel density. J Egypt Natl Canc Inst.

[31] Sanchez-Romero C, Bologna-Molina R, Mosqueda-Taylor A, de Almeida OP (2017). Immunohistochemical expression of podoplanin (D2-40), lymphangiogenesis, and neoangiogenesis in tooth germ, ameloblastomas, and ameloblastic carcinomas. J Oral Pathol Med.

[32] Yurugi Y, Wakahara M, Matsuoka Y, Sakabe T, Kubouchi Y, Haruki T, Nosaka K, Miwa K, Araki K, Taniguchi Y, Shiomi T, Nakamura H, Umekita Y (2017). Podoplanin Expression in Cancer-associated Fibroblasts Predicts Poor Prognosis in Patients with Squamous Cell Carcinoma of the Lung. Anticancer Res.

[33] Arigami T, Natsugoe S, Uenosono Y, Arima H, Mataki Y, Ehi K, Yanagida S, Ishigami S, Hokita S, Aikou T (2005). Lymphatic invasion using D2-40 monoclonal antibody and its relationship to lymph node micrometastasis in pN0 gastric cancer. Br J Cancer.

[34] Weidner N, Semple JP, Welch WR, Folkman J (1991). Tumor angiogenesis and metastasis—correlation in invasive breast carcinoma. N Engl J Med.

[35] Ozer E, Altungoz O, Unlu M, Aygun N, Tumer S, Olgun N (2007). Association of MYCN amplification and 1p deletion in neuroblastomas with high tumor vascularity. Appl Immunohistochem Mol Morphol.

[36] Sharma S, Sharma MC, Sarkar C (2005). Morphology of angiogenesis in human cancer: a conceptual overview, histoprognostic perspective and significance of neoangiogenesis. Histopathology.

[37] Korkolopoulou P, Apostolidou E, Pavlopoulos PM, Kavantzas N, Vyniou N, Thymara I, Terpos E, Patsouris E, Yataganas X, Davaris P (2001). Prognostic evaluation of the microvascular network in myelodysplastic syndromes. Leukemia.

[38] Shimada Y, Ishii G, Hishida T, Yoshida J, Nishimura M, Nagai K (2010). Extratumoral vascular invasion is a significant prognostic indicator and a predicting factor of distant metastasis in non-small cell lung cancer. J Thorac Oncol.

[39] Hishida T, Yoshida J, Maeda R, Ishii G, Aokage K, Nishimura M, Nagai K (2013). Prognostic impact of intratumoural microvascular invasion and microlymphatic permeation on node-negative non-small-cell lung cancer: which indicator is the stronger prognostic factor. Eur J Cardiothorac Surg.

[40] Pasquali S, van der Ploeg AP, Mocellin S, Stretch JR, Thompson JF, Scolyer RA (2013). Lymphatic biomarkers in primary melanomas as predictors of regional lymph node metastasis and patient outcomes. Pigment Cell Melanoma Res.

[41] Platt AM, Randolph GJ (2013). Dendritic cell migration through the lymphatic vasculature to lymph nodes. Adv Immunol.

[42] Swartz MA, Lund AW (2012). Lymphatic and interstitial flow in the tumour microenvironment: linking mechanobiology with immunity. Nat Rev Cancer.

[43] Wiig H, Swartz MA (2012). Interstitial fluid and lymph formation and transport: physiological regulation and roles in inflammation and cancer. Physiol Rev.

[44] Platt AM, Rutkowski JM, Martel C, Kuan EL, Ivanov S, Swartz MA, Randolph GJ (2013). Normal dendritic cell mobilization to lymph nodes under conditions of severe lymphatic hypoplasia. J Immunol.

[45] Heldin CH, Rubin K, Pietras K, Ostman A (2004). High interstitial fluid pressure-an obstacle in cancer therapy. Nat Rev Cancer.

[46] Bissanum R, Lirdprapamongkol K, Svasti J, Navakanitworakul R, Kanokwiroon K (2017). The role of WT1 isoforms in vasculogenic mimicry and metastatic potential of human triple negative breast cancer cells. Biochem Biophys Res Commun.

[47] Carmeliet P, Jain RK (2000). Angiogenesis in cancer and other diseases. Nature.

[48] Tong RT, Boucher Y, Kozin SV, Winkler F, Hicklin DJ, Jain RK (2004). Vascular normalization by vascular endothelial growth factor receptor 2 blockade induces a pressure gradient across the vasculature and improves drug penetration in tumors. Cancer Res.

[49] Fan Y, Du W, He B, Fu F, Yuan L, Wu H, Dai W, Zhang H, Wang X, Wang J, Zhang X, Zhang Q (2013). The reduction of tumor interstitial fluid pressure by liposomal imatinib and its effect on combination therapy with liposomal doxorubicin. Biomaterials.

[50] Paez-Ribes M, Allen E, Hudock J, Takeda T, Okuyama H, Vinals F, Inoue M, Bergers G, Hanahan D, Casanovas O (2009). Antiangiogenic therapy elicits malignant progression of tumors to increased local invasion and distant metastasis. Cancer Cell.

[51] Ribatti D (2011). Antiangiogenic therapy accelerates tumor metastasis. Leuk Res.

[52] Garcia-Caballero M, Paupert J, Blacher S, Van de Velde M, Quesada AR, Medina MA, Noel A (2017). Targeting VEGFR-3/-2 signaling pathways with AD0157: a potential strategy against tumor-associated lymphangiogenesis and lymphatic metastases. J Hematol Oncol.

[53] Carmeliet P, Jain RK (2011). Principles and mechanisms of vessel normalization for cancer and other angiogenic diseases. Nat Rev Drug Discov.

[54] Goel S, Wong AH, Jain RK (2012). Vascular normalization as a therapeutic strategy for malignant and nonmalignant disease. Cold Spring Harb Perspect Med.

[55] Jain RK, Tong RT, Munn LL (2007). Effect of vascular normalization by antiangiogenic therapy on interstitial hypertension, peritumor edema, and lymphatic metastasis: insights from a mathematical model. Cancer Res.

[56] Shimada H, Ambros IM, Dehner LP, Hata J, Joshi VV, Roald B, Stram DO, Gerbing RB, Lukens JN, Matthay KK, Castleberry RP (1999). The International Neuroblastoma Pathology Classification (the Shimada system). Cancer.

[57] Lejeune M, Lopez C, Bosch R, Korzynska A, Salvado MT, Garcia-Rojo M, Neuman U, Witkowski L, Baucells J, Jaen J (2011). JPEG2000 for automated quantification of immunohistochemically stained cell nuclei: a comparative study with standard JPEG format. Virchows Arch.

[58] Yamamoto G, Nannya Y, Kato M, Sanada M, Levine RL, Kawamata N, Hangaishi A, Kurokawa M, Chiba S, Gilliland DG, Koeffler HP, Ogawa S (2007). Highly sensitive method for genomewide detection of allelic composition in nonpaired, primary tumor specimens by use of affymetrix single-nucleotide-polymorphism genotyping microarrays. Am J Hum Genet.

[59] Ambros IM, Benard J, Boavida M, Bown N, Caron H, Combaret V, Couturier J, Darnfors C, Delattre O, Freeman-Edward J, Gambini C, Gross N, Hattinger CM (2003). Quality assessment of genetic markers used for therapy stratification. J Clin Oncol.

[60] Piqueras M, Navarro S, Canete A, Castel V, Noguera R (2011). How to minimise the effect of tumour cell content in detection of aberrant genetic markers in neuroblastoma. Br J Cancer.

[61] Villamon E, Berbegall AP, Piqueras M, Tadeo I, Castel V, Djos A, Martinsson T, Navarro S, Noguera R (2013). Genetic instability and intratumoral heterogeneity in neuroblastoma with MYCN amplification plus 11q deletion. PLoS One.

[62] Pan WR (2017). Atlas of Lymphatic Anatomy in the Head, Neck, Chest and Limbs.

[63] Raica M, Ribatti D, Mogoanta L, Cimpean AM, Ioanovici S (2008). Podoplanin expression in advanced-stage gastric carcinoma and prognostic value of lymphatic microvessel density. Neoplasma.

[64] Duff SE, Jeziorska M, Kumar S, Haboubi N, Sherlock D, O'Dwyer ST, Jayson GC (2007). Lymphatic vessel density, microvessel density and lymphangiogenic growth factor expression in colorectal cancer. Colorectal Dis.

[65] Tadeo I, Berbegall AP, Navarro S, Castel V, Noguera R (2017). A stiff extracellular matrix is associated with malignancy in peripheral neuroblastic tumors. Pediatr Blood Cancer.

