# Supplementary material for: Lymph microvascularization as a prognostic indicator in neuroblastoma
Source: Oncotarget. 2018 May 25;9(40):26157–70. doi: 10.18632/oncotarget.25457 (PMC5995242; doi:10.18632/oncotarget.25457)
Supplement: Supplementary file 1 [file oncotarget-09-26157-s001.pdf]

Lymph microvascularization as a prognostic indicator in neuroblastoma

SUPPLEMENTARY MATERIALS

Supplementary Table 1: Descriptors of the total vascularization and lymphatic vessel segments variables. See Supplementary_Table_1
