# Supplementary material for: Lymph microvascularization as a prognostic indicator in neuroblastoma
Source: Oncotarget. 2018 May 25;9(40):26157–70. doi: 10.18632/oncotarget.25457 (PMC5995242; doi:10.18632/oncotarget.25457)
Supplement: Supplementary file 2 [file oncotarget-09-26157-s002.docx]

**Supplementary Table 1: Descriptors of the total vascularization and lymphatic vessel segments variables**

| **Parameter** | **Mean** | **Median** | **Standard deviation** | **Minimum** | **Maximum** |
| --- | --- | --- | --- | --- | --- |
| **Total vascularization** | | | | | |
| Density | 161.1 | 102.4 | 177.7 | 0 | 1105 |
| Stained area | 1.7 | 1.2 | 1.9 | 0 | 15 |
| Average area | 100.9 | 69.6 | 278.9 | 22 | 4801 |
| Length | 13.9 | 12.5 | 13.1 | 8 | 239 |
| Width | 6.8 | 6.6 | 1.7 | 4 | 21 |
| Perimeter | 42.4 | 40 | 14.4 | 21 | 187 |
| Aspect | 2.2 | 2.1 | 0.2 | 2 | 4 |
| Roundness | 2.4 | 2.3 | 0.3 | 2 | 4 |
| Perimeter ratio | 0.84 | 0.85 | 0.01 | 0.79 | 0.90 |
| Deformity | 600.6 | 427.2 | 652.8 | 42 | 6198 |
| Shape factor | 2.2 | 0.4 | 21.5 | 0.1 | 373 |
| Branching | 2.7 | 2.7 | 0.2 | 2 | 4 |
| **Lymphatic vessel segments** | | | | | |
| *Small capillaries* | | | | | |
| Density | 121.1 | 84.1 | 130.9 | 0 | 735 |
| Stained area | 0.5 | 0.4 | 0.4 | 0 | 3 |
| Relative density | 67.6 | 76.1 | 27.4 | 0 | 100 |
| Relative stained area | 32.3 | 30.8 | 21.1 | 0 | 100 |
| Average area | 30.8 | 31.5 | 5.6 | 18 | 50 |
| Length | 8.6 | 8.6 | 0.4 | 7 | 10 |
| Width | 5 | 6.2 | 0.6 | 4 | 7 |
| Perimeter | 25.7 | 26 | 1.9 | 20 | 33 |
| Aspect | 2.1 | 2 | 0.3 | 2 | 4 |
| Roundness | 1.9 | 1.9 | 0.2 | 2 | 3 |
| Perimeter ratio | 0.87 | 0.87 | 0.008 | 0.84 | 0.91 |
| Deformity | 93.6 | 93.5 | 15.1 | 42 | 155 |
| Shape factor | 0.5 | 0.2 | 1.5 | 0.1 | 26 |
| Branching | 2.4 | 2.4 | 0.1 | 2 | 3 |
| *Intermediate capillaries* | | | | | |
| Density | 24.8 | 19.7 | 27 | 0 | 200 |
| Stained area | 0.52 | 0.31 | 0.61 | 0 | 4 |
| Relative density | 9.87 | 10.4 | 6.3 | 0 | 28 |
| Relative stained area | 24.7 | 27.6 | 13.7 | 0 | 60 |
| Average area | 196.3 | 191.3 | 57.7 | 84 | 768 |
| Length | 28.4 | 28.4 | 2.7 | 20 | 50 |
| Width | 12.8 | 12.8 | 2 | 7 | 27 |
| Perimeter | 95 | 94.6 | 13.7 | 64 | 210 |
| Aspect | 2.6 | 2.5 | 0.3 | 1 | 4 |
| Roundness | 4.1 | 4 | 0.76 | 2 | 14 |
| Perimeter ratio | 0.75 | 0.75 | 0.37 | 0.58 | 0.88 |
| Deformity | 1478.3 | 1456.2 | 398 | 356 | 3699 |
| Shape factor | 1.1 | 0.7 | 2 | 0.7 | 26 |
| Branching | 3.8 | 3.8 | 0.3 | 3 | 6 |
| *Large capillaries* | | | | | |
| Density | 18.5 | 14.7 | 19 | 0 | 143 |
| Stained area | 0.18 | 0.12 | 0.19 | 0 | 1 |
| Relative density | 7.8 | 8.5 | 4.1 | 0 | 23 |
| Relative stained area | 9.8 | 10.8 | 5.7 | 0 | 37 |
| Average area | 88 | 88.1 | 14.6 | 34 | 145 |
| Length | 17.1 | 17.2 | 0.8 | 16 | 19 |
| Width | 8.92 | 8.98 | 1.2 | 4 | 13 |
| Perimeter | 54.5 | 54.8 | 4.6 | 19 | 71 |
| Aspect | 2.3 | 2.2 | 0.4 | 1 | 6 |
| Roundness | 2.9 | 2.8 | 0.4 | 1 | 4 |
| Perimeter ratio | 0.79 | 0.79 | 0.04 | 0.41 | 0.90 |
| Deformity | 467 | 168.2 | 93 | 109 | 1004 |
| Shape factor | 0.74 | 0.44 | 1.2 | 0.1 | 13 |
| Branching | 3.26 | 3.29 | 0.3 | 1 | 4 |
